# Exploring the Current Applications of Artificial Intelligence in Orthopaedic Surgical Training: A Systematic Scoping Review

**DOI:** 10.7759/cureus.81671

**Published:** 2025-04-03

**Authors:** Ahmed Al-Saadawi, Sam Tehranchi, Syed Ahmed, Obinna J Nzeako

**Affiliations:** 1 School of Medicine and Dentistry, Queen Mary University of London, London, GBR; 2 Department of Trauma and Orthopaedic Surgery, Maidstone and Tunbridge Wells NHS Trust, London, GBR

**Keywords:** artificial intelligence, machine learning, medical education, orthopaedic surgery, surgical education

## Abstract

In recent years, the integration of artificial intelligence (AI) in surgical education has been prominent, as evidenced by recent publications. Given the unique requirements and challenges associated with orthopaedic training, we conducted a systematic scoping review that examined the applications of AI only in this setting. A comprehensive search was conducted across four databases: Embase, CENTRAL, Medline, and Scopus. Original research articles that utilised an AI model within a specific orthopaedic educational context were considered for inclusion. Data from the included studies were extracted onto a bespoke form, followed by a thematic analysis to detect patterns within the data. Our findings were then summarised descriptively. A total of 21 studies were included in the scoping review, encompassing 273 participants. In relation to the integration of AI in orthopaedic surgical training, two overarching themes were identified: refinement of surgical competencies and enhancement of knowledge acquisition. All included studies, with the exception of one, were conducted in the last five years. Twelve distinct AI models were utilised across the included studies, with large language models accounting for over half the applications. Multiple promising interventions were highlighted, particularly the use of personalised automated feedback models for evaluating performance in surgical tasks. AI holds major potential to revolutionise orthopaedic surgical training. However, evidence supporting its use in this field remains limited. Further studies, preferably randomised controlled trials with larger sample sizes, are required to strengthen the evidence base.

## Introduction and background

First introduced by Alan Turing in 1950, the concept of artificial intelligence (AI) was formally named in 1955 at a Dartmouth College summer workshop [1, 2]. It centres on technology that simulates intelligent behaviour and demonstrates problem-solving capabilities similar to those of humans [1]. AI does not refer to a singular model but is rather an encompassing term for countless subsets, each serving a unique role. One of the more popular subsets, machine learning (ML), revolves around computational algorithms that capture patterns within datasets and make accurate predictions based on this knowledge [3]. More recently, generative AI has spiked in popularity due to its remarkable ability to produce new, accurate content from simple prompts, enabling its use across a wide range of contexts [4]. With significant advancements in the last two decades, AI is now regarded as a future cornerstone in many key industries, especially healthcare [5].

In the healthcare setting, the use of AI is well documented [5]. It has demonstrated feasibility in improving diagnostic accuracy, predicting disease progression, enhancing patient outcomes, particularly in robotic surgery, and personalising patient care [6, 7]. Another component of healthcare where AI has become increasingly popular is medical education, especially surgical training [8]. Focusing on orthopaedics, the increasing number of complex procedures has limited opportunities for trainees to assist and develop their surgical skills. AI has demonstrated that it can overcome such challenges by enabling automated feedback systems that allow trainees to refine their skills in non-surgical training environments such as virtual reality (VR) [9]. Additionally, much of orthopaedic surgical training is based on the apprenticeship model, whereby trainees learn by observing their experienced mentor, replicating their actions, and subsequently receiving feedback on their performance [10]. While historically effective, the feedback can often be biased, limiting its overall usefulness to the trainee’s development [11]. With AI, particularly ML, the impartial assessment of trainee performance becomes a real possibility.

To our knowledge, several reviews have explored the use of AI in surgical education [12-14]. However, none have focused specifically on orthopaedics. Orthopaedic training is distinct from other surgical specialities, as it incorporates a broad skill set that covers multiple anatomical regions. With this in mind, we conducted a systematic scoping review that examined the applications of AI only in orthopaedic surgical training.

## Review

Methods

Protocol and Registration

This review was guided by Arksey and O’Malley’s framework [15], along with the modifications suggested by Levac et al. [16]. A description of the six stages within the framework is provided in Table 1. Throughout, we adhered to the PRISMA-ScR guidelines (Preferred Reporting Items for Systematic Reviews and Meta-Analyses extension for scoping reviews) [17] and the Joanna Briggs Institute (JBI) Manual for Evidence Synthesis. The protocol was registered with the Open Science Framework (OSF) on August 29, 2024 [18].

**Table 1:** Description of the six stages in Arksey and O’Malley’s framework for scoping reviews [15, 16]

Stage	Description
1	Identifying the research aims/questions
2	Identifying relevant studies
3	Study selection
4	Charting the data
5	Collating, summarizing and reporting results
6	Undertaking consultation

Identifying Relevant Studies

A comprehensive search was conducted across four databases: Medline, Embase, Scopus, and CENTRAL. These databases were searched from their inception to August 30, 2024. The search strategy employed for this review is outlined in Table 2 and was adapted to meet the requirements of each database. The primary keywords utilised in the strategy were “artificial intelligence”, “orthopaedic surgery”, and “medical education”. 

**Table 2:** Detailed search strategy employed for the scoping review

Section 1	Section 2	Section 3
exp Artificial Intelligence/ OR artificial* intelligence*.ti,ab. OR AI*.ti,ab. OR exp Machine Learning/ OR machine* learning*.ti,ab. OR neural* network*.ti,ab. OR exp Deep Learning/ OR deep* learning*.ti,ab. OR imag* adj2 recogni*.ti,ab. OR algorithm*.ti,ab. OR support vector machine*.ti,ab. OR SVM*.ti,ab. OR ChatGPT*.ti,ab. OR expNatural Language Processing/ OR natural language process*.ti,ab. OR	exp Orthopaedic Surgery/ OR exp Orthopedics/ OR orthopaedic*.ti,ab. OR orthopedic*.ti,ab. OR musculoskeletal*.ti,ab. OR trauma*.ti,ab. OR fractur*.ti,ab. OR arthtroplast*.ti,ab. OR arthroscop*.ti,ab. OR	exp Medical Education/ exp Education/ educat*.ti,ab. train*.ti,ab. learn*.ti,ab. skill*.ti,ab. reinforc*.ti,ab. residen*.ti,ab. (Section 1) AND (Section 2) AND (Section 3)

Study Selection

All articles were uploaded into a reference manager, where duplicate entries were identified and excluded. The remaining articles were subsequently uploaded onto Rayyan [19], and additional duplicates not detected by the reference manager were also excluded. Two independent reviewers (AS and ST) screened the studies on Rayyan, labelling each as *include*, *maybe*, or *exclude*. Articles that were labelled as *include* by both reviewers were considered for full-text review, whereas articles marked as *include* by one reviewer or *maybe* by both reviewers were discussed to reach a consensus. An independent third reviewer (ON) resolved any persisting disagreements. Additionally, the reference list of included studies was analysed to identify further relevant studies, which were then screened by both reviewers following the same screening process.

Inclusion Criteria

Population: Studies must either involve trainees in the research process or demonstrate a clear and direct impact on their education or training. The term *trainees* refers to orthopaedic residents, postgraduate residents, and/or medical students.

Concept: Studies were included if they applied artificial intelligence models with the aim of enhancing professional development.

Context: Studies were eligible if they were conducted within the setting of orthopaedic surgical training.

Study design: Original research articles that utilized AI models in the context of orthopaedic surgical training or education, and articles comparing AI interventions with a control group were included.

Exclusion Criteria

Editorials, abstracts, case reports, case series, expert opinion studies, posters, and review articles were excluded. Studies involving animal or cadaveric participants were excluded. Studies written in languages other than English were also excluded.

Charting the Data

Data from the included articles were extracted by two independent reviewers (AS and ST) using a bespoke form. The extracted variables included the author name, study date, study location, study design, population demographics, intervention characteristics, and main findings. Any disagreements in data extraction between the two reviewers were resolved through discussion. A third author (ON) served as an arbitrator if disagreements could not be resolved by consensus.

Collating, Summarising, and Reporting Results

Two independent reviewers (AS and ST) collated the extracted data into a pre-defined table in order to provide a visually clear and organised mapping of the existing evidence base. Following this, a thematic analysis was performed to detect patterns within the included studies and categorise them accordingly. The findings were summarised descriptively, detailing the aims, methodologies, and main conclusions of the included studies.

Undertaking Consultation

According to Arksey and O'Malley’s framework [15], this stage is considered optional within the context of scoping reviews. Due to time constraints, we were not able to conduct this stage in our review.

Results

**Figure 1:**
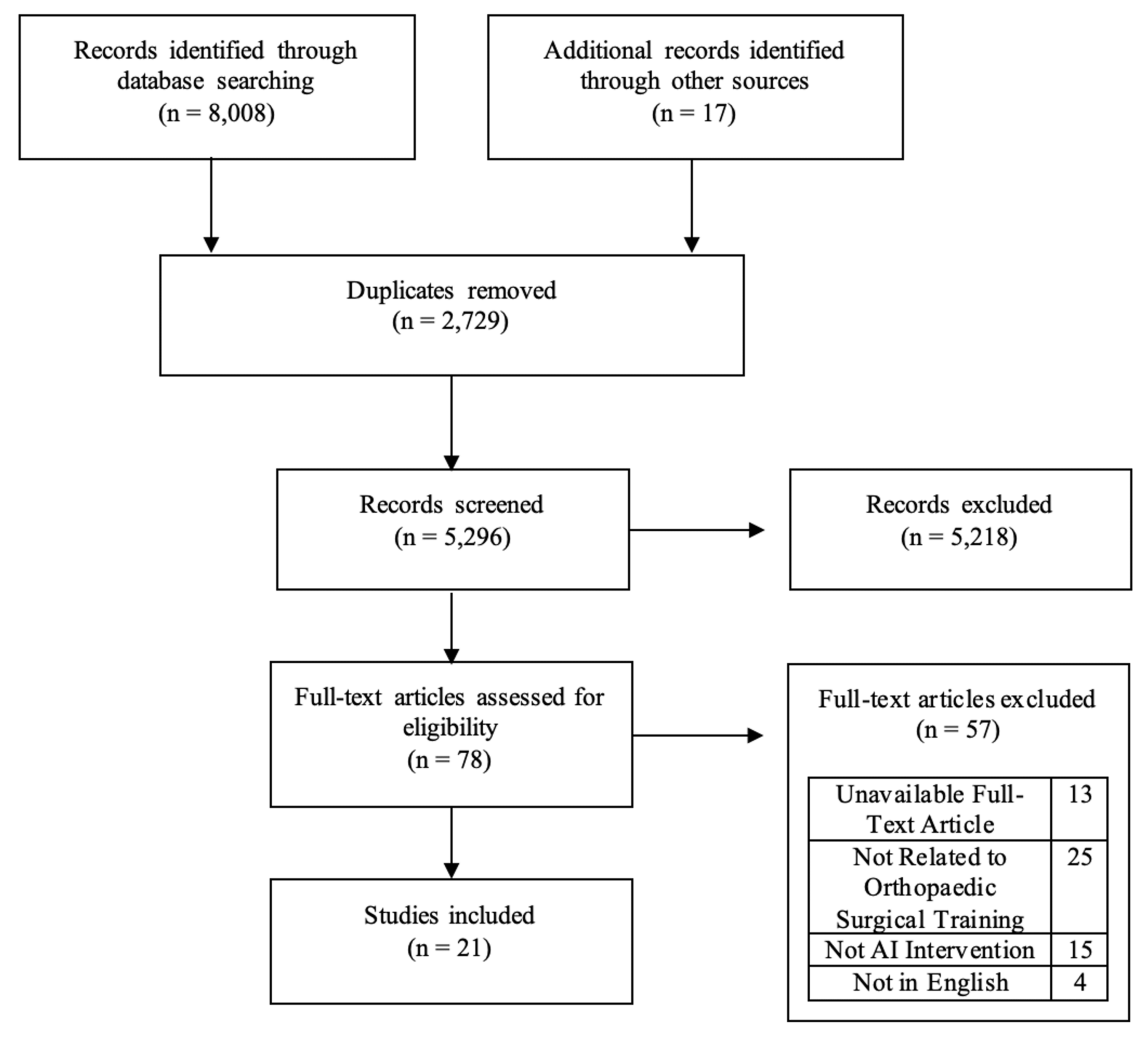
PRISMA flowchart illustrating selection of studies PRISMA: Preferred Reporting Items for Systematic Reviews and Meta-Analyses.

The search identified 8,025 articles, which were reduced to 5,296 after the removal of duplicates. Following the screening of titles and abstracts, 78 articles were selected for full-text review. Of these, 25 studies were excluded because they lacked relevance to orthopaedic surgical training, and an additional 15 studies were excluded because they did not integrate AI into the intervention design. Ultimately, 21 studies met the inclusion criteria and were included in the final review (Figure 1).

Characteristics of Included Studies

Twenty-one studies were included in the final review [20-41], involving a total of 273 participants. The studies were carried out in various locations, including the United States of America (n = 11), Canada (n = 6), Germany (n = 1), France (n = 1), Taiwan (n = 1), China (n = 1), and South Korea (n = 1). Most of the studies were published in the last five years (2019 to 2024) [20-39, 41], with the exception of one from 2017 [40].

Three studies [20, 24, 27] included cohorts exclusively composed of trainees. In contrast, eight studies [21, 26, 30, 32, 36, 39-41] included a mixture of trainees and experienced surgeons to evaluate whether their respective AI models could discriminate between varying expertise levels. One study [35] used “academic staff” as their cohort but did not provide further details on their qualifications. Eleven studies [22, 23, 25, 28, 29, 31, 33, 34, 37-39] did not include a human cohort.

A total of 12 different AI interventions were utilised: large language models (n = 12), convolutional neural network (n = 4), artificial neural networks (n = 2), support vector machine (n = 2), K-nearest neighbour (n = 2), linear discriminant analysis (n = 2), decision trees (n = 1), recurrent neural network (n = 1), Naïve Bayes network (n = 1), long short-term memory (n = 1), K-means clustering algorithm (n = 1), and an unspecified neural network (n = 1). A summary of the included studies can be seen in Table 3. Across the studies, two main themes were identified related to the integration of AI in orthopaedic surgical training: refinement of orthopaedic surgical skills and enhancement of knowledge acquisition.

**Table 3:** Summary of included studies RCT: randomised controlled trial, ♂: male, ♀: female, AI: artificial intelligence, PXR: posterior X-ray, DL: deep learning, ChatGPT: Chat Generative Pre-trained Transformer, MLP-ANN: multilayer perceptron artificial neural network, NLP: natural language processing, SVM: support vector machine, KNN: k-nearest neighbour, LDA: learning discriminant analysis, DT: decision trees, NB: naïve Bayes, CNN: convolutional neural network, RNN: recurrent neural network, LSTM: long short-term memory, KMC: k-means clustering.

Study/Setting	Study Design	Population Characteristics	AI Model	Intervention Characteristics	Main Findings
Cheng et al., 2020, Taiwan [20]	RCT	Sample size: Experimental: 15 (9 ♂ and 6 ♀); Control: 15 (10 ♂ and 5 ♀). • Mean age: Experimental: 24 ± 1.60; Control: 24.67 ± 3.13. • Level of expertise: Fifth-year medical students	CNN (DenseNet-121)	Experimental: To generate heatmaps on PXR images to enhance fracture detection learning. • Control: Participants received standard PXR images for hip fracture detection learning, without AI assistance.	The experimental group showed a significant improvement in the post-learning fracture detection assessment compared to the control group (P = 0.042).
Alkadri et al., 2022, Canada [21]	Validation	Sample size: Junior Resident: 7 (5 ♂ and 2 ♀); Senior Resident: 5 (4 ♂ and 1 ♀); Post-resident: 11 (11 ♂ and 0 ♀). • Mean age: Junior Resident: 27.4 ± 1.4; Senior Resident: 30.6 ± 2.3; Post-resident: 44.2 ± 13.2. • Level of expertise: Junior Resident, Senior Resident, Post-resident	MLP-ANN	To analyse performance and provide feedback in a simulated annulus task.	The ANN model achieved an 80% testing accuracy.
Ozdag et al., 2023, USA [22]	Experimental	Sample size: N/A. • Mean age: N/A. • Level of expertise: N/A	LLM (ChatGPT 3.5)	To assess its performance on 102 questions from the US Orthopaedic In-Training Examination.	ChatGPT correctly answered 46 out of 102 questions (45%).
Lum et al., 2023, USA [23]	Experimental	Sample size: N/A. • Mean age: N/A. • Level of expertise: N/A	LLM (ChatGPT – Unspecified version)	To measure its performance on 207 questions from the US Orthopaedic In-Training Examination.	ChatGPT answered 97 questions correctly (47%).
DeCook et al., 2024, USA [24]	Experimental	Sample size: 3. • Mean age: N/A. • Level of expertise: Orthopaedic surgical trainees	LLM (ChatGPT 4.0)	Experimental: To produce educational summaries on total knee arthroplasty. • Control: Trainees were tasked with creating educational summaries on total knee arthroplasty.	ChatGPT outperformed orthopaedic trainees in creating orthopaedic content across all domains combined (P < 0.001). • ChatGPT generated educational summaries at a significantly quicker pace than orthopaedic trainees (P = 0.02)
Massey et al., 2023, USA [25]	Experimental	Sample size: N/A. • Mean age: n/a. • Level of expertise: N/A	LLM (ChatGPT 3.5 + 4.0)	To evaluate and compare their performance to orthopaedic residents on 180 questions from the Orthopaedic Assessment Examination.	Orthopaedic residents scored 72.4% on the examination, significantly higher than the 29.4% and 47.2% achieved by ChatGPT 3.5 and 4, respectively (P < 0.001).
Bissonnette et al., 2019, Canada [26]	Validation	Sample size: Senior: 22; Junior: 19. • Mean age: N/A. • Level of expertise: Senior - spine surgeon, fellows, and senior residents; Junior: Junior residents and medical students	SVM, KNN, LDA, DT, NB	To differentiate between levels of surgeon expertise in a virtual reality hemilaminectomy task.	The SVM model was most successful with an accuracy of 97.6%.
Meetschen et al., 2024, Germany [27]	Validation	Sample size: 4. • Mean age: N/A. • Level of expertise: Postgraduate residents	DCNN	To aid learning in radiographic fracture detection.	The DCNN model achieved a sensitivity of 93% and a specificity of 77%. • The model significantly reduced radiograph interpretation time (P = 0.0156) and enhanced confidence among postgraduate residents (P = 0.0013)
Isleem et al., 2023, USA [28]	Experimental	Sample size: N/A. • Mean age: N/A. • Level of expertise: N/A	LLM (ChatGPT – Unspecified version)	To measure its performance on the US Orthopaedic In-Training Examination.	ChatGPT 4.0 correctly answered 183 out of 301 questions (60.8%).
Kim et al., 2024, South Korea [29]	Experimental	Sample size: N/A. • Mean age: N/A. • Level of expertise: N/A	LLM (ChatGPT 3.5 + 4.0)	To assess their performance on 160 text-only questions from the Korean Orthopaedic Association Board Examination.	ChatGPT 3.5 answered 37.5% of questions correctly (60/160). • ChatGPT 4.0 answered 60% of questions correctly (96/160).
Casy et al., 2022, France [30]	Prospective cohort	Sample size: Novice: 36 (26 ♂ and 10 ♀); Intermediate: 12 (11 ♂ and 1 ♀); Expert: 12 (11 ♂ and 1 ♀). • Mean age: Novice: 25.17; Intermediate: 32.5; Expert: 56.72. • Level of expertise: Novice: Junior residents; Intermediate: < 5 years since obtaining a postgraduate diploma in orthopaedic surgery; Expert: > 5 years since obtaining a postgraduate diploma in orthopaedic surgery	CNN (OpenPose)	To analyse and distinguish postural behaviour across different levels of surgeon expertise in an arthroscopic simulation.	No significant difference (P > 0.05) was observed in the mean mobility score between novice (0.823), intermediate (0.816), and expert (0.820) orthopaedic surgeons.
Han et al., 2024, USA [31]	Experimental	Sample size: N/A. • Mean age: N/A. • Level of expertise: N/A	LLM (ChatGPT 3.5)	To test its performance on the American Society for Surgery of the Hand self-assessment examinations from 2014 to 2023.	ChatGPT 4.0 delivered its best performance on the 2012 self-assessment examination, with an overall score of 42.2%.
Demirel et al., 2022, USA [32]	Validation	Sample size: Novice: 2; Expert: 2. • Mean Age: N/A. • Level of expertise: Novice: Surgeons with extensive orthopaedic residency training; Expert: Surgeons who have undergone fellowship programs for rotator cuff procedures	CNN	To provide automated quantitative assessments of performance in rotator cuff surgeries.	The DL model achieved a test accuracy ranging from 85 to 95%.
Fekri et al., 2020, Canada [33]	Experimental	Sample size: N/A. • Mean age: N/A. • Level of expertise: N/A	RNN, LSTM	To provide real-time haptic guidance to optimize skill transfer during femoral drilling simulation procedures.	The model delivered appropriate haptic-guidance signals in simulation, accurately replicating the behaviour of expert orthopaedic surgeons.
Ghanem et al., 2024, USA [34]	Experimental	Sample size: N/A. • Mean age: N/A. • Level of expertise: N/A	LLM (ChatGPT 4.0)	To test its performance on the 2019 American Hand Surgery Certifying Examination.	ChatGPT 4.0 achieved an overall score of 61.98%.
Mirchi et al., 2022, Canada [36]	Validation	Sample size: Junior Resident: 7 (5 ♂ and 2 ♀); Senior Resident: 5 (4 ♂ and 1 ♀); Post-resident: 9 (9 ♂ and 0 ♀). • Mean age: Junior Resident: 27.4 ± 1.4; Senior Resident: 30.6 ± 2.3; Post-resident: 44.2 ± 13.2. • Level of expertise: Junior Resident; Senior Resident; Post-resident	ANN	To quantify and assess surgical expertise in a simulated vertebral osteophyte removal task.	The ANN model demonstrated a testing accuracy of 83.3%.
Fiedler et al., 2024, USA [37]	Experimental	Sample size: N/A. • Mean age: N/A. • Level of expertise: N/A	LLM (ChatGPT 3.5 + 4.0)	To assess and compare its performance on the 2023 American Shoulder and Elbow Surgeon self-assessment examination with that of fellowship-trained surgeons.	Fellowship-trained orthopaedic surgeons outperformed ChatGPT 4.0 on image-based questions (P = 0.019), whereas their performance on text-based questions did not differ significantly (P = 0.136). • Across all questions, fellowship-trained orthopaedic surgeons demonstrated significantly superior performance compared to ChatGPT 4.0 (75.3% vs. 60.2%, P = .012).
Jain et al., 2024, USA [38]	Experimental	Sample size: N/A. • Mean age: N/A. • Level of expertise: N/A	LLM (ChatGPT 3.5)	To evaluate its performance on the US Orthopaedic In-Service Training Examinations.	ChatGPT 4.0 recorded its best score on the 2020 examination, with a score of 55.8%.
Rizzo et al., 2023, USA [39]	Experimental	Sample size: N/A. • Mean age: N/A. • Level of expertise: N/A	LLM (ChatGPT 3.5 + 4.0)	To assess their performance on the US Orthopaedic In-Training Examinations.	ChatGPT 4.0 outperformed ChatGPT 3.5 across all exams. • ChatGPT 4.0 achieved its highest score on the 2022 examination, with a score of 67.63%.
Poursartip et al., 2017, Canada [40]	Validation	Sample size: Novice: 18; Expert: 8. • Mean age: N/A. • Level of expertise: Novice: Orthopaedic residents, surgeons without prior scoping experience, and non-surgeons; Expert: Fellowship-trained orthopaedic surgeons	SVM, NN, KNN, LDA	To evaluate motor skill proficiency in arthroscopic simulation through the use of an energy expenditure metrics system.	The NN and LDA models were the most successful, achieving accuracy rates between 80 and 95%.
Sun et al., 2021, China [41]	Validation	Sample size: 58 (56 ♂ and 2 ♀); Beginner: 21 (20 ♂ and 1 ♀); Mid-level: 17 (16 ♂ and 1 ♀); Experienced: 19 (19 ♂ and 0 ♀). • Mean age: 43.4. • Level of expertise: Licensed orthopaedic surgeons with varying levels of experience	KMC	To assess whether differences in gap balancing ability in cadaveric unicompartmental knee arthroplasty exist between varying surgeon expertise levels.	The experienced group achieved a significantly lower mean difference between extension and flexion forces compared to the mid-level (P = 0.023) and beginner group (P < 0.001).

Refinement of Orthopaedic Surgical Skills

Eight studies [21, 26, 30, 32, 33, 36, 40, 41] assessed the use of AI as a tool to improve proficiency in orthopaedic surgical tasks. Of these, seven studies [21, 26, 30, 32, 36, 40, 41] used ML algorithms to develop an automated feedback system that analysed performance and subsequently provided personalised feedback to surgeons. Alternatively, to optimise surgical skill transfer, Fekri et al. proposed a deep learning algorithm designed to provide real-time haptic guidance signals to novice surgeons, modelling the behaviour of senior orthopaedic surgeons [33]. The assessed orthopaedic surgical tasks included annulus incision [21], hemilaminectomy [26], ergonomics (during arthroscopy) [30], rotator cuff repair [32], femoral bone drilling [33], vertebral osteophyte removal [36], shoulder arthroscopy [40], and knee arthroplasty [41]. Six studies [21, 26, 30, 33, 36, 40] utilised virtual reality (VR) simulation environments to conduct these tasks, while one analysed surgical video recordings [32] and another used cadaveric specimens [41]. 

The most commonly used metrics to assess surgical performance included dynamics such as force and velocity [21, 26, 33, 36, 41], anatomical content removal [21, 26, 36], and tool motion and usage [26, 32, 33, 36]. Poursartip et al. incorporated various forms of energy, including mechanical energy and work, into their metrics to evaluate motor skill proficiency in arthroscopic surgery [40]. Studies using VR to assess the aforementioned surgical tasks generated these metrics directly from the simulator [21, 26, 33, 36, 40], except for Casy et al. [30], who evaluated ergonomic performance by tracking the frequency of joint movements using an RGB camera. 

Bissonnette et al. introduced several ML models to evaluate performance in a simulated hemilaminectomy, with their support vector machine achieving the highest test accuracy at 97.6% [26]. Four other studies reported similar success, with accuracies ranging from 80% to 96% [21, 32, 36, 40]. Sun et al. reported that their K-means clustering algorithm successfully distinguished gap balancing ability after unicompartmental knee arthroplasty between experienced surgeons (83.5 ± 73.8 Newtons (N)) and mid-level (214.7 ± 115.4 N, P = 0.023) or beginner surgeons (346.4 ± 162.1 N, P < 0.001) [41]. However, no significant difference was observed between mid-level and beginner surgeons (P = 0.113) [41]. Differing from the other models, Alkadri et al. incorporated a unique component in their algorithm by employing a multilayer network to enable calculation of the Connection Weights Product (CWP) [21]. This quantifies the significance of each metric in discriminating between surgeon expertise levels, thereby highlighting crucial areas for trainee surgeons to improve on. For example, the CWP pinpointed that C4 contact length and duration were the main metrics differentiating junior residents from senior and post-residents [21]. Fekri et al.’s deep neural network model also demonstrated promising potential, accurately predicting haptic force feedback [33]. Additionally, Casy et al. reported that their deep learning algorithm was unable to detect significant differences in ergonomic performance between varying surgeon expertise levels [30]. 

Enhancing Orthopaedic Knowledge Acquisition

Thirteen studies [20, 22-25, 27-29, 31, 34, 37-39] assessed the potential of AI as a tool to enhance orthopaedic knowledge acquisition in trainees. Ten studies [22, 23, 25, 28, 29, 31, 34, 37-39] utilised Chat Generative Pre-Trained Transformer (ChatGPT), a large language model, to answer orthopaedic residency exam questions, assessing its usefulness in supporting orthopaedic resident education and exam preparation. Ghanem et al. reported that ChatGPT 4.0 scored 61.98% on the American Hand Surgery Board Certification Examination, successfully meeting the passing threshold [34]. Four other studies [25, 29, 37, 39] used ChatGPT 4.0, achieving scores ranging from 47.2% to 67.63%. Conversely, ChatGPT 3.5, the older counterpart, demonstrated poorer performance, with seven studies reporting scores ranging from 29.4% to 55.8% [22, 25, 29, 31, 37-39]. Both Lum et al. [23] and Isleem et al. [28] did not specify the version of ChatGPT used in their studies, achieving scores of 47% and 60.8%, respectively. Considering their respective performances and publication dates (Lum et al.: 2023; Isleem et al.: 2024), it is likely that Lum et al. [23] utilised ChatGPT 3.5, while Isleem et al. [28] used version 4.0. Four studies [22, 25, 34, 37] compared their findings with historical data on resident performance in the corresponding examinations, each revealing that orthopaedic residents significantly outperformed either version of ChatGPT. 

Two studies [20, 27] presented ML algorithms to accelerate the learning process for radiographic fracture detection among trainees. Cheng et al. used a convolutional neural network to generate heatmaps on radiographs to aid fifth-year medical students in detecting hip fractures [20]. Students who used AI-assisted radiographs in their learning exhibited significantly greater improvements in accuracy from pre-learning to post-learning assessment (9.2 ± 6.9) compared to their unaided peers (2.8 ± 9.3, P = 0.042) [20]. Meetschen et al. evaluated the effectiveness of a deep convolutional neural network to assist postgraduate residents in detecting adult and paediatric trauma fractures on radiographs [27]. With the support of AI, postgraduate residents experienced improved fracture detection (P < 0.0001), reduced interpretation time (P = 0.0156), and increased confidence in findings (P = 0.0013) [27].

DeCook et al. evaluated whether ChatGPT 4.0 is more effective at generating professional educational content on total knee arthroplasty compared to orthopaedic fellows [24]. ChatGPT produced superior educational content compared to orthopaedic fellows when all domains were combined (P ≤ 0.001). Additionally, ChatGPT demonstrated impressive speed, producing educational summaries in just 16 seconds compared to the 94 minutes required by orthopaedic fellows (P = 0.006) [24].

Discussion

This systematic scoping review gathered and examined the existing literature on the applications of AI in orthopaedic surgical training. To the best of our knowledge, this is the first scoping review that specifically focuses on the applications of AI in orthopaedic surgical training. A diverse range of models were utilised, with large language models and neural networks accounting for most applications. Their integration into orthopaedic training centred on two core themes: refinement of orthopaedic surgical skills and enhancement of knowledge acquisition. Overall, the scoping review highlighted several promising interventions, including personalised automated feedback models for evaluating surgical performance and the efficient production of orthopaedic educational material.

In the United Kingdom, the Trauma & Orthopaedic (T&O) Surgery curriculum is built on a competency-based system, where trainees must fulfil a pre-defined set of core skills to progress in their training [42]. Despite this, there is thought to be a lack of opportunities for orthopaedic trainees, particularly in surgical settings, to assume roles with increased intra-operative responsibility, hindering their overall clinical and professional development [43-45]. Simulation training offers a potential solution to this issue by allowing trainees to practise surgical skills in a risk-free environment [46]. However, the assessment of performance in simulation training is typically quantified using the Objective Structured Assessment of Technical Skills (OSATS) tool, which requires experienced examiners, of whom there is a limited number [9]. In this review, six automated feedback models [21, 26, 32, 36, 40, 41] achieved accuracies of at least 80%, underscoring their strong ability to differentiate between novice and expert surgeon performance, and in turn, provide targeted feedback that addresses the weaknesses of novice orthopaedic surgeons. However, a key criticism of simulation training is that it lacks the anatomical variability that cadaveric models can provide [47]. In this context, it may not offer an entirely realistic and transferable training experience. A possible solution to this issue is the integration of human digital twins (virtual models that leverage AI and real-time data to replicate patients) into simulation training [47, 48]. Initially proposed by Dean et al., this technology can incorporate patient-specific features into VR simulation models, rather than relying on a generic anatomical model, enabling greater surgical adaptability and ultimately enhancing patient outcomes [47].

If effectively applied, AI has the potential to be a transformative tool for orthopaedic surgical training. It enables trainees to develop their skills outside regular training hours, without the need for educators, and often from a distance, personalising their learning experience to meet individual needs and thereby enhancing their development [49]. Additionally, it can help standardise orthopaedic training across the UK, where regional disparities in training opportunities exist [50]. For example, McAlinden et al. report that tendon repairs, an index procedure in the Trauma & Orthopaedic Surgery curriculum, are often performed by plastic surgeons in Northern Ireland, limiting opportunities for orthopaedic trainees to gain the necessary experience [50]. On a larger scale, AI can be leveraged to address the challenges associated with orthopaedic training in middle- and low-income countries. Its capacity to provide more accessible, cost-effective, and improved learning opportunities will hopefully raise the number of trained, competent orthopaedic surgeons in these regions, which is a crucial necessity [49].

Although promising, the integration of AI in orthopaedic training is still novel, and several major challenges need to be addressed before it can be adopted into everyday practice. First, there are ethical implications that must be considered, especially regarding patient privacy [51]. Developing AI models for major healthcare applications, such as medical education, requires significant data to ensure unbiased and accurate decision-making. Meeting this requirement often means that multiple institutions must work together, which can be challenging, as patient data may have to be shared [52]. Robust guidelines relevant to healthcare data sharing must be developed to ensure that patient confidentiality is preserved amid the continuing rise of AI.

Another challenge is the potential opposition from educators to integrating AI into orthopaedic training, due to concerns that it may eventually render their jobs obsolete [53]. Despite these fears, this is an unrealistic expectation, as orthopaedic training heavily relies on mentorship and guidance, areas where AI still struggles to provide true value.

Limitations

Although our search strategy was well-developed, it focused on identifying studies with “artificial intelligence” or related terms in the title and abstract. As a result, we may have inadvertently missed studies that met our inclusion criteria but did not explicitly mention the use of AI in these sections. Furthermore, the studies included in our review were predominantly of a validation or experimental design and, therefore, did not assess whether these AI applications had a measurable impact on the development of orthopaedic trainees. In this context, it was difficult to evaluate the true effectiveness of these models in orthopaedic training, ultimately underscoring the need for further research with longer follow-up periods to assess their long-term feasibility and impact.

## Conclusions

Overall, this scoping review has highlighted several promising AI applications in orthopaedic surgical training, such as personalised automated feedback models for evaluating surgeon performance. However, the evidence supporting its use remains limited, as the field is still emerging and major obstacles must be addressed before wider implementation is feasible. Future research with larger sample sizes and longer follow-up periods is needed to build upon the findings outlined in this review.
